# High incidence of *Trypanosoma cruzi* infections in dogs directly detected through longitudinal tracking at 10 multi-dog kennels, Texas, USA

**DOI:** 10.1371/journal.pntd.0009935

**Published:** 2021-11-10

**Authors:** Rachel E. Busselman, Alyssa C. Meyers, Italo B. Zecca, Lisa D. Auckland, Andres H. Castro, Rebecca E. Dowd, Rachel Curtis-Robles, Carolyn L. Hodo, Ashley B. Saunders, Sarah A. Hamer

**Affiliations:** 1 Department of Veterinary Integrative Biosciences, Texas A&M University, College Station, Texas, United States of America; 2 Department of Epidemiology, School of Public Health, University of Michigan, Ann Arbor, Michigan, United States of America; 3 The University of Texas MD Anderson Cancer Center, Michale E. Keeling Center for Comparative Medicine and Research, Bastrop, Texas, United States of America; 4 Department of Small Animal Clinical Sciences, Texas A&M University, College Station, Texas, United States of America; FDA: US Food and Drug Administration, UNITED STATES

## Abstract

Canine Chagas disease, caused by the protozoan parasite *Trypanosoma cruzi*, is increasingly recognized as a health concern for dogs in the USA, and infected dogs may signal geographic regions of risk for human disease. Dogs living in multi-dog kennel environments (kennels with more than one dog) where triatomine vectors are endemic may be at high risk for infection. We monitored a cohort of 64 *T*. *cruzi*-infected and uninfected dogs across 10 kennels in Texas, USA, to characterize changes in infection status over one year. We used robust diagnostic criteria in which reactivity on multiple independent platforms was required to be considered positive. Among the 30 dogs enrolled as serologically- and/or PCR-positive, all but one dog showed sustained positive *T*. *cruzi* diagnostic results over time. Among the 34 dogs enrolled as serologically- and PCR-negative, 10 new *T*. *cruzi* infections were recorded over a 12-month period. The resulting incidence rate for dogs initially enrolled as *T*. *cruzi-*negative was 30.7 *T*. *cruzi* infections per 100 dogs per year. This study highlights the risk of *T*. *cruzi* infection to dogs in kennel environments. To protect both dog and human health, there is an urgent need to develop more integrated vector control methods as well as prophylactic and curative antiparasitic treatment options for *T*. *cruzi* infection in dogs.

## Author summary

Chagas disease is a deadly parasitic heart disease of humans, dogs, and other mammals that is spread by kissing bug vectors. In order to understand the risk of Chagas parasite infection to dogs over time, we followed infected and uninfected dogs (n = 64) to monitor changes in their status. This work took place across ten different large dog kennels in Texas, where there was a history of vector presence and canine deaths due to Chagas disease. In the absence of a gold standard diagnostic test, multiple parasite and antibody detection methods were used. Direct re-sampling of dogs over a 12-month period showed that all but one positive dog maintained the infection, and nearly one-third of the dogs that started the study as negative had converted to positive. Otherwise stated, a dog at these kennels has a risk of 30.7% of acquiring *T*. *cruzi* infection within one year. Novel vector control methods, antiparasitic treatment approaches, and diagnostic tests for Chagas disease are urgently needed to protect both animal and human health.

## Introduction

The protozoan parasite *Trypanosoma cruzi*, agent of Chagas disease, is a vector-borne zoonotic pathogen endemic to the Americas. According to estimates based on 2010 data, over 5.7 million people were infected with *T*. *cruzi* in 21 Latin American countries in addition to hundreds of mammal species [1,2]. *T*. *cruzi* is predominantly transmitted in the feces of infected triatomines (‘kissing bugs’) through contact with wounds or mucous membranes or ingestion of infected insects or fecal material [1]. Oral transmission is thought to be the most important route in domestic dogs and wild mammals and is a highly efficient mode of transmission [3–6].

Enzootic cycles of *T*. *cruzi* transmission have been documented in the southern USA, where opossums (*Didelphis virginiana*), woodrats (*Neotoma* spp.), coyotes (*Canis latrans*), and other mammals naturally infected with *T*. *cruzi* come into contact with endemic triatomine species [7–9] and maintain parasite transmission [1,10–13]. The wildlife species involved in the transmission of *T*. *cruzi* often inhabit peridomestic environments and share spaces with domestic dogs outdoors, increasing dogs’ risk of exposure to infected vectors [11,14]. In settings where dogs share spaces with humans in and around homes, dogs serve as sentinels for human infection, maintaining *T*. *cruzi* transmission and amplifying disease in domestic and peridomestic environments [15,16]. Thus, areas of high *T*. *cruzi* seroprevalence in domestic dogs may indicate areas of heightened risk for human infection [17].

In the USA, *T*. *cruzi* infection has been reported in dogs across the southern states [18–20]. Studies from Texas, Oklahoma, and Louisiana report dog infection prevalence from 3.6–22.1%, and up to 57.6% in some multi-dog kennels, or kennels with more than one dog [20–28]. Infection with *T*. *cruzi* shows no strong breed predilection, and shelter and stray dogs are likely exposed to *T*. *cruzi* often due to their high level of exposure to vectors, resulting in high prevalence of infection [20,23,29,30]. Additionally, government working dogs along the USA-Mexico border are exposed to *T*. *cruzi* with a seroprevalence of up to 18.9% [27], in which infections have led to fatal cardiac disease [31]. Canine Chagas diagnostics are challenged by the limited number of approved tests validated for use in dogs in the USA, and often, in accordance with WHO guidelines for Chagas testing in humans, more than one serologic test is required for canine Chagas diagnoses. In both humans and dogs, Chagas disease causes a range of clinical symptoms, progressing through acute and chronic stages of disease, which can include severe heart disease and death [4,22,32]. While insect vectors are endemic to the southern USA, canine travel introduces a veterinary health concern as infected dogs move outside of endemic areas to areas where clinicians are less familiar with the symptoms of Chagas disease [33].

While measures of infection prevalence from cross-sectional studies are useful in quantifying the burden of disease in populations, the incidence rate (i.e. the number of new infections per population per unit time) can provide a direct measurement of risk. Direct measurements of incidence in natural animal populations are rare, as they require tracking of healthy, uninfected individuals over time to monitor for new infections. Alternatively, incidence can be inferred indirectly by comparing seroprevalence across different age cohorts of animals. Using this indirect approach based on serology, we are aware of two studies investigating the *T*. *cruzi* incidence rate in dog populations in the USA. The first estimated a serologic incidence of 3.8% in juvenile dogs along the Texas-Mexico border [34]; the second estimated an incidence rate of 2.3 new cases per year in dogs of all ages in Louisiana shelters [20]. We used a longitudinal study design to directly measure incidence of *T*. *cruzi* infection in dogs of Central/South Texas, an area with a high risk of infection based on suitable triatomine habitat and autochthonous human cases [35]. Uncertainty about transmission risk and the lack of an effective treatment or vaccine pose significant problems to veterinarians and dog owners following diagnoses of Chagas disease. In this study, we tracked the serostatus and PCR-status of a matched cohort of *T*. *cruzi-*positive and negative dogs at three intervals over 12-months, allowing for the direct detection of *T*. *cruzi* infection in dogs in kennel environments.

## Methods

### Ethics statement

All samples were obtained from privately-owned animals in accordance with guidelines approved by the Texas A&M University’s Institutional Animal Care and Use Committee and Clinical Research Review Committee (2018–0460 CA).

### Locations and sample collection

Using a prospective longitudinal study design, we enrolled a network of 10 multi-dog kennels throughout Central and South Texas with a prior history of *T*. *cruzi* infection in at least one of their dogs. Kennel locations were categorized by Texas ecoregions [29]. Dogs at these kennels were generally year-round residents of the kennel, typically leaving the kennel for hunting or training during the day; dogs at one kennel spent some months in the summer in a northern state to train. They were primarily bred and trained to aid hunting parties or compete in American Kennel Club dog events and were classified according to American Kennel Club breed groups. Enrollment criteria included dogs of any breed or sex at least one year of age, in residence at one of the selected kennels, and negative for other selected vector-borne infectious diseases (*Anaplasma* spp., *Ehrlichia* spp., *Borrelia burgdorferi*, and *Dirofilaria immitis*).

We aimed to identify and enroll approximately two to four *T*. *cruzi*-infected and two to four *T*. *cruzi*-uninfected dogs at each kennel for longitudinal tracking at approximate 6-month intervals. During the initial sampling time point between May and July 2018, we tested 134 dogs (between seven and 20 dogs at each of the 10 kennels) for evidence of *T*. *cruzi* and four other vector-borne infections. Based on the enrollment criteria, 64 dogs were identified as meeting the criteria and were enrolled in the study. When possible, *T*. *cruzi-*positive and *T*. *cruzi-*negative dogs were frequency (group) matched on the basis of age, sex, and breed across all kennels. Each blood sampling event consisted of collecting approximately 5mL of blood in an EDTA (ethylenediaminetetraacetic acid) tube and 4mL in a clotting tube. The second sampling event occurred between December 2018 and March 2019 (4.7–9.2 months, average = 6.3 months; 6-month Time Point), and the third sampling event occurred between May and September 2019 (5.4–7.4 months, average = 6.4 months; 12-month Time Point). All 10 kennels were visited at each time point to collect blood samples from enrolled dogs.

### Serology

At each of the three time points, serum samples were tested for *T*. *cruzi* antibodies using three serological tests: Chagas Stat-Pak (ChemBio, Medford, NY, USA), Chagas *Detect Plus* Rapid Test (InBios International, Inc., Seattle, WA, USA), and an indirect fluorescent antibody (IFA) test. The Chagas Stat-Pak and Chagas *Detect Plus* are rapid tests that, while not labeled for use in dogs, have been shown to be sensitive and specific to detect *T*. *cruzi* antibodies and are commonly used in dogs for research purposes [26,36,37]. Serum samples were tested according the manufacturer’s instructions, and results were determined after 15 minutes. In all cases, the integrated ‘control’ bands appeared as expected on the rapid tests. Rapid tests were considered negative when the ‘test’ band had no color development or when a very faint band- not perceptible enough to be a clear positive- developed. The IFA was performed by the Texas A&M Veterinary Medical Diagnostic Laboratory (TVMDL) using 200 μL of serum to test for IgG antibodies against *T*. *cruzi*. Reactivity on at least two of the three serologic tests was one of the two possible criteria (in addition to PCR positivity, see below) to consider a dog *T*. *cruzi-*positive for enrollment. An IDEXX 4Dx test (IDEXX Laboratories, Inc., Westbrook, ME, USA) was run at the time of enrollment to exclude any dogs that may have had other vector-borne infections, including *Anaplasma* spp., *Ehrlichia* spp., *Borrelia burgdorferi*, and *Dirofilaria immitis*.

### DNA extraction, quantification, and PCR

A DNA extraction procedure from large volumes (5mL) of EDTA-treated blood was used in an effort to increase sensitivity of detection of *T*. *cruzi* using the E.Z.N.A Blood DNA Maxi kit (Omega Bio-Tek, Norcross, GA, USA) according to the manufacturer’s protocol, except 650μL of elution buffer was used. *T*. *cruzi-n*egative controls (phosphate buffered saline) were included in the DNA extractions. For each extracted sample, DNA was quantified using a spectrophotometer (Epoch, BioTek Instruments, Inc., Winooski, VT, USA). In the case that any errors had occurred during the extraction process, as seen by low DNA yield from the MAXI extraction (<85 ng/μL) or control wells that suggested contamination, a second extraction was performed using 250μL of clotted blood using the E.Z.N.A. Tissue DNA kit, according to the manufacturer’s protocol, except 50 μL of elution buffer was used (Omega Bio-Tek).

Samples were tested using qPCR for the presence of *T*. *cruzi* satellite DNA using the Cruzi 1/2 primer set and Cruzi 3 probe in a real-time assay, which amplifies a 166-bp segment of a repetitive nuclear DNA [38,39] as previously described [27]. This PCR method is sensitive and specific for *T*. *cruzi* when compared to other PCR techniques (sensitivity = 83.3, specificity = 95; compared with kDNA and Satellite DNA PCRs as described in [40]. *T*. *cruzi* DNA extracted from isolate Sylvio X10 CL4 (ATCC 50800, American Type Culture Collection [ATCC], Manassas, VA, USA; DTU TcI) was used as the *T*. *cruzi-*positive control in all PCRs. The qPCR amplification curves were checked for quality (sigmoidal curves), and a sample was considered *T*. *cruzi-*positive if the Ct value was lower than 36 [41].

### Statistical analyses

Dogs were categorized as *T*. *cruzi*-positive at a given time point if they met the criteria for serological positivity (positive on at least two of three independent serologic assays) and/or if they met the criteria for PCR positivity (Ct value lower than 36). We calculated the Kappa index to determine the agreement among all three serological tests [20,27]. To describe the risk of *T*. *cruzi* infection in the population of dogs that were initially enrolled as *T*. *cruzi-*negative, we calculated the incidence rate as the number of new *T*. *cruzi* infections per 100 dogs per year. To reflect the time *T*. *cruzi-*negative dogs in the population were at risk, 0.5 years was subtracted from the population at risk (n = 34) for each of the three *T*. *cruzi-*negative dogs lost to follow up after the 6-month time point (n = 3).

## Results

### Dog enrollment

Of the 134 dogs screened, we enrolled 30 *T*. *cruzi-*positive and 34 *T*. *cruzi-*negative dogs across 10 kennels in Central and South Texas (Table 1). Kennels were located in one of four ecoregions, primarily (7/10) in the South Texas Plains. Dogs enrolled in the study were between the ages of 13 months and 12.2 years (mean = 6.1 years; median = 6.1 years). Dogs were frequency matched based on age, sex, and breed: age was matched within 8 months (average *T*. *cruzi-*positive = 6.6 years; average *T*. *cruzi-*negative = 5.8 years), sex was matched within four dogs (positive = 14 males and 16 females; negative = 19 females and 15 males), and breeds were matched between *T*. *cruzi-*positive and *T*. *cruzi-*negative dogs (various hound breeds were considered “Hound” for matching purposes; three positive and negative Belgian Malinois, two positive and negative German Shorthair Pointers, three positive and negative Hounds, one positive and seven negative Labrador Retrievers, two positive and negative Brittany Spaniels, and 19 positive and 17 negative English Pointers per group). During the initial screening of 134 dogs across all 10 kennels, one dog was positive for *Dirofilaria immitis* and was excluded from the study. In total, five dogs were lost to follow up after the 6-month time point: two dogs enrolled as *T*. *cruzi-*negative (including one that seroconverted to positive by 6-months) moved locations and were sold to new owners, and three dogs (one *T*. *cruzi-*positive and two negative) had died.

**Table 1:** Location, demographic data, and *T*. *cruzi* infection status of dogs at the time of enrollment, with data on new *T*. *cruzi* infections.

			Dog enrollment status			
Variable		Total # dogs enrolled	*T*. *cruzi*-positive	*T*. *cruzi*-negative	# dogs converted from negative to positive	Dogs lost to follow-up
Location by Ecoregion						
	Gulf Prairie	8	4	4	1 (25%)	0
	South Texas Plains	42	19	23	6 (26%)	4
	Edwards Plateau	6	3	3	0	0
	Post Oak Savannah	8	4	4	3 (75%)	1
Sex						
	Male	31	16	15	6 (40%)	3
	Female	33	14	19	4 (21%)	2
Breed						
	Belgian Malinois	6	3	3	2 (67%)	0
	Hound	6	3	3	0	0
	Brittany Spaniel	4	2	2	2 (100%)	0
	English Pointer	36	19	17	5 (29%)	4
	German Shorthaired Pointer	4	2	2	1 (50%)	1
	Labrador Retriever	8	1	7	0	0
Age (years)						
	≤6	32	13	19	5 (26%)	1
	>6	32	17	15	5 (33%)	4

Of the 30 dogs enrolled as *T*. *cruzi-*positive at the initial time point, 26 (86.7%) were positive on all three serologic tests; two (6.7%) were positive on both rapid tests yet negative on IFA; and one (3.3%) was positive on a single rapid test and had a high titer (>1280) on IFA (Table 2). Additionally, one dog (D76) was enrolled as *T*. *cruzi-*positive based solely on PCR positivity (3.3%; Table 2). Ten of the aforementioned *T*. *cruzi-*seropositive dogs were also PCR positive. The overall prevalence of PCR-positivity at the time of enrollment among dogs enrolled as *T*. *cruzi-*positive was 11/30 (36.7%; Table 3).

**Table 2:** Serology of the 30 dogs enrolled as *T*. *cruzi-*positive in the study. The two rapid tests (Chagas Stat-Pak and Chagas *Detect Plus*) are scored as Positive (“+”) or Negative (“-”), and the IFA titer is reported in which a result of <20 is noted as Negative (“-”).

	Initial Time Point			6-month Time Point			12-month Time Point		
Dog ID	Chagas Stat-Pak	IFA Titer	Chagas *Detect Plus*	Chagas Stat-Pak	IFA Titer	Chagas *Detect Plus*	Chagas Stat-Pak	IFA Titer	Chagas *Detect Plus*
D05	+	5120	+	+	640	+	+	1280	+
D06	+	5120	+	+	2560	+	+	1280	+
D07	+	2560	+	+	160	+	+	640	+
D08	+	2560	+	+	640	+	+	2560	+
D17	+	**-**	+	+	**-**	+	+	**-**	+
D21	+	5120	+	+	2560	+	+	640	+
D26	+	320	+	+	160	+	+	160	+
D27	+	2560	+	+	640	+	+	5120	+
D30	+	**-**	+	+	**-**	+	+	**-**	+
D39	+	160	+	+	**-**	+	+	20	+
D42	+	640	+	+	160	+	+	640	+
D43	+	640	+	+	320	+	+	320	+
D49	+	640	+	+	>1280	+	+	320	+
D54	+	80	+	+	160	+	+	80	+
D75	+	640	+	+	320	+	+	5120	+
D76*	-	-	-	-	-	-	-	-	-
D77	+	1280	+	+	320	+	+	5120	+
D79	+	2560	+	+	320	+	+	5120	+
D94	+	1280	+	+	80	+	+	640	+
D96	+	2560	+	+	640	+	+	5120	+
D99	+	1280	+	+	320	+	+	320	+
D100	+	640	+	+	160	+	+	320	+
D103	+	1280	+	+	160	+	+	640	+
D104	+	1280	+	+	640	+	+	2560	+
D108	+	2560	+	+	640	+	+	5120	+
D110	+	320	+	+	160	+	+	320	+
D121	+	320	+	+	320	+	+	160	+
D122	+	320	+	+	160	+	+	80	+
D123	+	80	+	+	40	+	+	160	+
D124	**-**	>1280	+	**-**	640	+		Deceased	

*D76 was serologically-negative but enrolled as *T*. *cruzi-*positive due to PCR positivity in the blood.

**Table 3:** PCR results (Ct values) for dogs with a Ct value indicating a *T*. *cruzi-*positive sample. No Ct value or a Ct value of 36 or higher during qPCR is considered a negative PCR result and is noted as “-” in this table.

Dog ID	Initial Time Point	6-month Time Point	12-month Time Point
D06	34.6	-	-
D07	31.4	-	-
D76†	33.2	35.5	-
D21	26.7	29.4	26.0
D27	29.7	32.6	28.7
D43	33.5	30.1	29.2
D79	32.5	25.1	25.1
D108	25.6	27.3	23.5
D42	31.1	-	31.8
D100	26.4	-	28.9
D104	18.6	-	30.2
D125*†	-	-	29.4
D131*	-	-	15.7
D97*	-	-	17.0

* Dog enrolled as *T*. *cruzi-*negative

† Was serologically-negative at the time point with a positive PCR result

Of the 34 dogs enrolled as *T*. *cruzi-*negative, 31 (91.2%) were negative on all three serologic tests and PCR at the initial time point. Three dogs (3/34, 8.8%) were weakly positive on a single rapid test (two dogs were positive on only the Chagas Stat-Pak, and one dog was positive only on the Chagas *Detect Plus*).

### DNA extraction

Eluted DNA concentrations from MAXI extractions used in the study ranged from 88.6 ng/μL to 348.5 ng/μL (n = 104; mean = 172.4 ng/μL; median = 164.2 ng/μL).

### Longitudinal tracking

Twenty-nine of the 30 dogs enrolled as *T*. *cruzi-*positive maintained their *T*. *cruzi-*positive status at the 6-month and 12-month time points across the study with minor fluctuations in individual serologic and PCR test results over time; for example, one dog (D39) was positive on all three serological assays during the initial and 12-month time point but positive on only the two rapid tests during the 6-month time point (Tables 2 and 3). The dog enrolled as *T*. *cruzi-*positive based on PCR-positivity only (D76) was again PCR-positive at the 6-month time point but PCR-negative at the 12-month time point and never developed antibodies detectable on any of the three serologic assays. Of the 11 dogs enrolled as *T*. *cruzi-*positive that had PCR-positive results at the initial time point, five dogs (45.4%) were consistently PCR-positive at all three time points, 3 (27.3%) were PCR-positive at only the first and third sampling trips, one dog (9.1%) was PCR-positive at only the first and second visits, and two dogs (18.2%) were only PCR-positive at the initial time point (Table 3).

**Fig 1:**
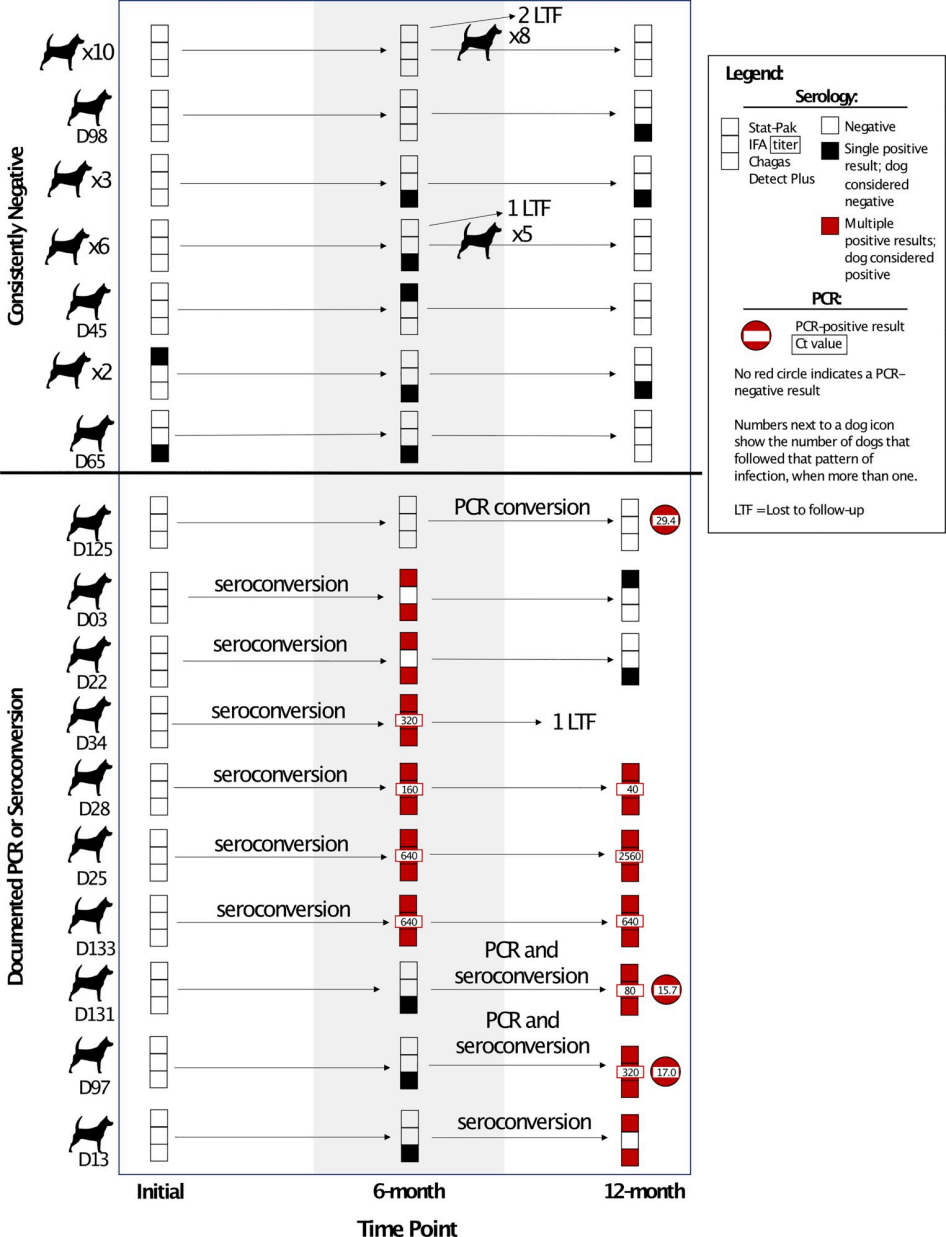
Serology and PCR results of all dogs enrolled as *T*. *cruzi-*negative (n = 34) at each sampling time point. Dogs are organized by those that remained negative above the black horizontal line and those that had a documented PCR or seroconversion during the study below the line. When multiple dogs had the same progression of test results, this progression is depicted as a single row in the figure with the number of dogs shown on the left side of the figure. Changes in serostatus or PCR status are written between time points in which the conversion took place. The titer for positive IFA tests and the Ct value for PCR-positive tests are noted in the respective symbol.

Of the 34 dogs enrolled as *T*. *cruzi-*negative, 24 (70.6%) remained negative at the 6-month and 12-month time points (Fig 1). One dog (2.9%) remained serologically-negative but was PCR-positive at the 12-month time point, and nine dogs (26.5%) were seropositive by the end of the study; therefore, these 10 dogs met the study definition of *T*. *cruzi* positive (Fig 1). Notably, two dogs (5.9%) were seropositive on both rapid serologic assays at the 6-month time point and therefore met the definition for seroconversion, but were seropositive on only one rapid serologic assay by the 12-month time point. In total, three dogs enrolled as *T*. *cruzi-*negative (8.8%) were PCR-positive at the 12-month time point (Fig 1).

The Kappa Indices comparing serological tests across all time points showed the Chagas Stat-Pak and IFA had almost perfect agreement (kappa = 0.84), while there was substantial agreement between the Chagas *Detect Plus* and IFA and Chagas *Detect Plus* and Chagas Stat-Pak (kappa = 0.65 and 0.70 respectively) (Table 4).

### Incidence

A total of 10 out of the 34 (29.4%) dogs enrolled as *T*. *cruzi-*negative converted to positive throughout the one-year study. After adjusting the time the total population was at risk to account for attrition of three *T*. *cruzi-*negative dogs after the 6-month time point, this results in an incidence rate of 30.7 *T*. *cruzi* infections per 100 dogs per year. If the two dogs that seroconverted by the 6-month time point but only maintained positivity on a single test at the 12-month time point (D03 and D22) were not true conversions, then the incidence rate would be 24.6 new *T*. *cruzi* infections per 100 dogs per year.

## Discussion

We characterized the *T*. *cruzi* serologic and PCR-status of 64 dogs at 3 time points over the course of one year and demonstrated a remarkably high incidence of *T*. *cruzi* infection in dogs housed in multi-dog kennels across Texas. In our cohort, 34 dogs were negative at enrollment, and we recorded 10 new infections (29.4%) resulting in an incidence rate of 30.7 new infections per 100 dogs per year. Otherwise stated, a dog at these kennels has a risk of 30.7% of acquiring *T*. *cruzi* infection within one year. Enrollment of dog kennels was biased toward kennels that had a known history of Chagas disease, and so results may not be generalizable to other kennel environments where triatomines do not occur and those without a history of canine Chagas disease. However, triatomines are reported broadly from Texas and many southern states [42], and it is likely that canine Chagas disease is under-detected and underreported.

Direct quantification of *T*. *cruzi* incidence in dogs is rare, likely due to the challenges of collecting longitudinal data [43]. More commonly, incidence is estimated indirectly. For example, researchers conducted a serosurvey of 540 dogs in Louisiana and found a 6.9% prevalence of *T*. *cruzi* infection [20]. Assuming exposure to *T*. *cruzi* infection is similar between animals and over time, they also estimated the incidence of 2.3 new infections per 100 dogs/year. This estimate is similar to that of another study, where researchers estimated the incidence in juvenile dogs along the Texas-Mexico border [34]. In this second study, the *T*. *cruzi* prevalence in juvenile dogs under 6 months old was used to estimate a *T*. *cruzi* serologic incidence of 3.8%, assuming all infections that occurred in dogs under 6 months old were newly acquired infections. This methodology does not consider congenital infections, and further, can only be applied to the juvenile age group. In contrast to these previous studies estimating incidence to be much lower than our calculated incidence, our longitudinal study allowed us to directly detect new *T*. *cruzi* infections using serology and PCR over one year, and thus report a direct measure of incidence in a closed population of dogs.

There is no gold standard to test for *T*. *cruzi* infections in dogs, and the IFA is widely used in clinical practice across the USA for veterinary Chagas diagnostics. While the Chagas Stat-Pak and the Chagas *Detect Plus* are often used for research purposes, discordant test results are common in canine testing for Chagas disease [27]. As recommended by the WHO for human Chagas diagnostics [44], reactivity on multiple serologic assays was the criteria used in our study to consider a dog *T*. *cruzi-*positive. We observed substantial to high agreement across serologic tests, reflecting the strict inclusion criteria and matched study design, where *T*. *cruzi-*positive dogs were enrolled as positive only when there was some agreement across serologic assays. Overall, the Chagas Stat-Pak and IFA had the best agreement with an overall kappa index of 0.84 (Table 4); whereas, there was reduced pairwise agreement between the other combinations of tests (kappa range 0.65–0.70). Cross-sectional studies of dog *T*. *cruzi* serostatus have found less agreement, even high discordance, between Chagas tests using the Kappa index, with only slight to moderate agreement between tests [20,27]. Given the absence of gold standard antemortem testing methods, our data support the WHO recommendation that a multi-test approach is still necessary in dog Chagas disease diagnostics. However, none of our selected serologic assays could identify IgM antibodies, which have been used to detect acute antibody responses against *T*. *cruzi* in dogs [16,45] and could have been correlated with PCR data. PCR was used in addition to serologic tests as a diagnostic tool for *T*. *cruzi* in this study, with the ability to detect new infections prior to antibody formation. The PCR protocol used in this study was shown to be one of the most sensitive and specific PCR methods to detect *T*. *cruzi* based on an international survey of PCR methods of *T*. *cruzi* detection [40] and has been previously used to detect *T*. *cruzi* in blood samples [39]. We expect to see fewer PCR positives than antibody positive results because PCR-positivity indicates active circulation of the parasite in the animals’ blood, which may occur for only short time periods in infected dogs [4]. For example, D42, D100, and D104 were all PCR-positive at the initial time point and final (12-month) time point yet were PCR-negative at the 6-month time point (Table 3). This variation in PCR results over time may be due to transient parasitemia from an initial infection, or due to re-infections [46], as our data show *T*. *cruzi* is readily infecting dogs in these areas. By using four independent tests to categorize animals as *T*. *cruzi-*positive or negative, and repeating sampling at 3 time points to look for change in infection status, our study design was robust and sensitive to capture new infections in a way that has not before been done in veterinary Chagas studies in the United States.

**Table 4:** Kappa Index table to statistically show pairwise comparisons and agreement among three serological tests: Chagas Stat-Pak, Chagas *Detect Plus*, and indirect fluorescent antibody (IFA) test.

A.	IFA+	IFA -	Total
Chagas Stat-Pak +	86	13	99
Chagas Stat-Pak -	2	86	88
Total	88	99	187
Almost perfect agreement:	**0.84**		
B.	IFA+	IFA -	
Chagas *Detect Plus* +	88	33	121
Chagas *Detect Plus* -	0	66	66
Total	88	99	187
Substantial agreement:	**0.65**		
C.	Chagas Stat-Pak +	Chagas Stat-Pak -	
Chagas *Detect Plus* +	96	25	121
Chagas *Detect Plus* -	3	63	66
Total	99	88	187
Substantial agreement:	**0.70**		

In this study, one dog (D76) was PCR-positive yet antibody negative at enrollment, indicating the presence of *T*. *cruzi* DNA in the blood at the time of sampling, and thus this dog was enrolled as *T*. *cruzi-*positive. We expected this dog to develop detectable antibodies by the time of the 6- and 12-month time points. However, the dog did not seroconvert and displayed a weak PCR-positive result (high-positive Ct value) at the 6-month time point. The reasons for a lack of seroconversion in this dog are unknown but could be due to health conditions that resulted in a suppressed immune system or other health measures we did not assess as part of this study, or due to a false positive PCR result.

Two dogs (D03 and D22) met the definition of incident cases because of their initial enrollment as *T*. *cruzi-*negative (each had one very faint band on one test; see Methods; classified as negative) and seroconversion at the 6-month time point, at which time they were reactive on both rapid serologic assays. However, at the 12-month time point, both dogs were only positive on one serologic assay (D03 on the Chagas Stat-Pak; D22 on the Chagas *Detect Plus*), and therefore failed to meet the diagnostic criteria for positivity at that time point, which required reactivity on at least two independent tests. As a new infection followed by self-cure would be unlikely [1,43], we suspect this scenario reflects the limitations of using unvalidated diagnostic tests for dogs, and future prospective sampling from these dogs would be enlightening. If these two dogs represent false conversions, this means our incidence rate would be lower, at 24.6 new infections per 100 dogs/year. Even an incidence only considering eight new infections (out of 34) is markedly higher than previously estimated dog incidence rates for *T*. *cruzi* infections. Additionally, the three dogs enrolled as negative, despite having a weakly positive band on a single rapid test, remained negative through the course of the study with either one weakly positive test or no positive test at each timepoint, providing evidence in this study that a single weak band is not likely to signal an early infection or an infection that will become more diagnostically apparent in the next year. The need for improved diagnostic tools for detection of *T*. *cruzi* infection is apparent for human medicine [47], and our study highlights the need in veterinary medicine as well [27].

Over the course of the study, five dogs were lost to follow up either due to death or to moving locations. Unfortunately, necropsies were not performed and postmortem samples were not available; thus, the causes of death (and potential role of Chagas disease) remain unknown. One of the deceased dogs was *T*. *cruzi-*positive, and the owners described a slow decline over two months with observed weight loss, but no veterinary visits or treatment were elected by the owners.

There was geographic variation in the frequency of incident cases across the 10 studied kennels. Kennels varied from no incident infections to three of the four dogs enrolled as *T*. *cruzi-*negative having newly acquired *T*. *cruzi* infection. The incident cases were distributed across six of the 10 kennels. This variation may indicate natural variation in triatomine abundance or infection among kennels [42], or the importance of other factors, such as vector control around the kennels, kennel integrity, or additional exposure to kissing bugs while hunting or training outside of the kennels, such as on other areas of the owners’ property.

There is no validated treatment or prevention for all stages of Chagas disease in dogs, and diagnosis often occurs during the chronic phase when experimental treatments rarely change the outcome of disease and treatment is designed to manage symptoms [4]. As no vaccines exist for dogs and cardiac symptoms can cause acute death or chronic illness [48], preventing contact with infected vectors is paramount; this requires a thorough understanding of transmission cycles and risk. Accordingly, Chagas disease prevention and control is largely focused on reducing a dog’s contact with vectors. Approaches to halting contact include vector control with insecticides applied to kennel outdoor spaces, keeping dogs indoors, avoiding outdoor lights that attract bugs, and altering vegetation to reduce the density of wildlife reservoirs and kissing bugs. However, various vector control measures are challenging, as no insecticides are specifically labeled for use in the control of triatomines in the USA. In particular, it is common for adult kissing bugs to be encountered in kennel environments, posing a transmission risk to the dogs [42]. Triatomines have proven difficult to eradicate even in areas with robust vector control programs, as locating the bugs is difficult, time consuming, and sometimes unsuccessful for species that colonize the domestic environment [49,50]. Widespread vector control campaigns in the USA have not been attempted for triatomines, owing to their predominantly sylvatic occurrence [42] with occasional spillover to domestic settings.

Multi-dog kennels should be areas of targeted intervention in the USA, as the incidence of dog infection is high. We found dogs at many of these kennels have a high risk of *T*. *cruzi* infection over the course of one year, highlighting the need to develop thorough vector control methods that can be deployed in kennel environments and identify, test, and make available prevention and treatment options for *T*. *cruzi* infection in dogs in the USA. The loss of hunting and working dogs has an economic impact on breeders, trainers, and ranch owners, and developing next steps to prevent or treat canine Chagas disease is not only necessary for veterinary health but to decrease the human impact of Chagas disease as well. Further, travel of infected dogs from endemic regions of the southern USA to non-endemic regions where there is less awareness of Chagas disease is occurring [33], in which case diagnosis may be harder to attain. Similarly, in northern Mexico, canine *T*. *cruzi* infections are also common, with seroprevalence estimates of 4.4% and 9.5% in communities near the USA border [45,51]. Differences in the level of canine infection among studies may relate to differences in the local abundance of infected vectors that feed on dogs and/or may relate to different diagnostic approaches. Because dogs can achieve a level of parasitemia sufficient to infect kissing bugs during acute disease, there is concern for infected dogs serving as a local reservoir to perpetuate the parasite life cycle [23,25,52]. Accordingly, any interventions to reduce infection in dogs and improve their overall health may also have the added benefit of reducing the risk of locally acquired human disease as well.

## Supporting information

S1 Strobe Checklist STROBE Statement—Checklist of items that should be included in reports of *cohort studies*. (DOCX)

## References

[1] Bern C, Kjos S, Yabsley MJ, Montgomery SP. *Trypanosoma cruzi* and Chagas’ disease in the United States. Clin Microbiol Rev. 2011;24(4):655–81. doi: 10.1128/CMR.00005-11 21976603 PMC3194829

[2] World Health Organisation. 2015. Chagas disease in Latin America: an epidemiological update based on 2010 estimates. Wkly Epidemiol Rec 2015; 90: 33–43. 25671846

[3] Roellig DM, Ellis AE, Yabsley MJ. Oral transmission of *Trypanosoma cruzi* with opposing evidence for the theory of carnivory. J Parasitol. 2009;95(2):360–4. doi: 10.1645/GE-1740.1 18763853 PMC2911628

[4] Barr SC. Canine Chagas’ disease (American trypanosomiasis) in North America. Vet Clin North Am Small Anim Pract. 2009;39(6):1055–64, v-vi. doi: 10.1016/j.cvsm.2009.06.004 19932362

[5] Velasquez-Ortiz N, Ramirez JD. Understanding the oral transmission of *Trypanosoma cruzi* as a veterinary and medical foodborne zoonosis. Res Vet Sci. 2020;132:448–61. doi: 10.1016/j.rvsc.2020.07.024 32781335

[6] Montenegro VM, Jimenez M, Dias JC, Zeledon R. Chagas disease in dogs from endemic areas of Costa Rica. Mem Inst Oswaldo Cruz. 2002;97(4):491–4. doi: 10.1590/s0074-02762002000400006 12118277

[7] Kjos SA, Snowden KF, Olson JK. Biogeography and *Trypanosoma cruzi* infection prevalence of Chagas disease vectors in Texas, USA. Vector Borne Zoonotic Dis. 2009;9(1):41–50. doi: 10.1089/vbz.2008.0026 18800865

[8] Curtis-Robles R, Wozniak EJ, Auckland LD, Hamer GL, Hamer SA. Combining public health education and disease ecology research: Using citizen science to assess Chagas disease entomological risk in Texas. PLoS Negl Trop Dis. 2015;9(12):e0004235. doi: 10.1371/journal.pntd.0004235 26658425 PMC4687635

[9] Zecca IB, Hodo CL, Slack S, Auckland L, Hamer SA. *Trypanosoma cruzi* infections and associated pathology in urban-dwelling Virginia opossums (*Didelphis virginiana*). Int J Parasitol Parasites Wildl. 2020;11:287–93. doi: 10.1016/j.ijppaw.2020.03.004 32215248 PMC7090276

[10] Curtis-Robles R, Lewis BC, Hamer SA. High *Trypanosoma cruzi* infection prevalence associated with minimal cardiac pathology among wild carnivores in central Texas. Int J Parasitol Parasites Wildl. 2016;5(2):117–23. doi: 10.1016/j.ijppaw.2016.04.001 27330982 PMC4900435

[11] Hodo CL, Hamer SA. Toward an ecological framework for assessing reservoirs of vector-borne pathogens: Wildlife reservoirs of *Trypanosoma cruzi* across the southern United States. ILAR J. 2017;58(3):379–92. doi: 10.1093/ilar/ilx020 29106561 PMC6019048

[12] Gunter SM, Brown EL, Gorchakov R, Murray KO, Garcia MN. Sylvatic transmission of *Trypanosoma cruzi* among domestic and wildlife reservoirs in Texas, USA: A review of the historical literature. Zoonoses Public Health. 2017;64(5):313–27. doi: 10.1111/zph.12330 27911051

[13] Bern C, Montgomery SP. An estimate of the burden of Chagas disease in the United States. Clin Infect Dis. 2009;49(5):e52–4. doi: 10.1086/605091 19640226

[14] Burkholder JE, Allison TC, Kelly VP. *Trypanosoma cruzi* (Chagas) (Protozoa: Kinetoplastida) in invertebrate, reservoir, and human hosts of the lower Rio Grande valley of Texas. J Parasitol. 1980;66(2):305–11. 6771371

[15] Gurtler RE, Cardinal MV. Reservoir host competence and the role of domestic and commensal hosts in the transmission of *Trypanosoma cruzi*. Acta Trop. 2015;151:32–50. doi: 10.1016/j.actatropica.2015.05.029 26051910

[16] Estrada-Franco JG, Bhatia V, Diaz-Albiter H, Ochoa-Garcia L, Barbabosa A, Vazquez-Chagoyan JC, et al. Human *Trypanosoma cruzi* infection and seropositivity in dogs, Mexico. Emerg Infect Dis. 2006;12(4):624–30. doi: 10.3201/eid1204.050450 16704811 PMC3294681

[17] Jaimes-Duenez J, Triana-Chavez O, Cantillo-Barraza O, Hernandez C, Ramirez JD, Gongora-Orjuela A. Molecular and serological detection of *Trypanosoma cruzi* in dogs (*Canis lupus familiaris*) suggests potential transmission risk in areas of recent acute Chagas disease outbreaks in Colombia. Prev Vet Med. 2017;141:1–6. doi: 10.1016/j.prevetmed.2017.03.009 28532988

[18] Valdez-Tah A, Ibarra-Cerdena CN. Call to action: A literature review of Chagas disease risk in California 1916–2018. PLoS Negl Trop Dis. 2021;15(2):e0009035. doi: 10.1371/journal.pntd.0009035 33630839 PMC7906329

[19] Rodriguez F, Luna BS, Calderon O, Manriquez-Roman C, Amezcua-Winter K, Cedillo J, et al. Surveillance of *Trypanosoma cruzi* infection in triatomine vectors, feral dogs and cats, and wild animals in and around El Paso county, Texas, and New Mexico. PLoS Negl Trop Dis. 2021;15(2):e0009147. doi: 10.1371/journal.pntd.0009147 33600455 PMC7924784

[20] Elmayan A, Tu W, Duhon B, Marx P, Wolfson W, Balsamo G, et al. High prevalence of *Trypanosoma cruzi* infection in shelter dogs from southern Louisiana, USA. Parasit Vectors. 2019;12(1):322. doi: 10.1186/s13071-019-3572-y 31238941 PMC6593594

[21] Bradley KK, Bergman DK, Paul Woods J, Crutcher JM, Kirchhoff LV. Prevalence of American trypanosomiasis (Chagas disease) among dogs in Oklahoma. JAVMA. 2000;217(12):1853–7. doi: 10.2460/javma.2000.217.1853 11132891

[22] Kjos SA, Snowden KF, Craig TM, Lewis B, Ronald N, Olson JK. Distribution and characterization of canine Chagas disease in Texas. Vet Parasitol. 2008;152(3–4):249–56. doi: 10.1016/j.vetpar.2007.12.021 18255233

[23] Beard CB, Pye G, Steurer FJ, Rodriguez R, Campman R, Peterson AT, et al. Chagas disease in a domestic transmission cycle, southern Texas, USA. Emerg Infect Dis. 2003;9(1):103–5. doi: 10.3201/eid0901.020217 12533289 PMC2873735

[24] Hodo CL, Rodriguez JY, Curtis-Robles R, Zecca IB, Snowden KF, Cummings KJ, et al. Repeated cross-sectional study of *Trypanosoma cruzi* in shelter dogs in Texas, in the context of *Dirofilaria immitis* and tick-borne pathogen prevalence. J Vet Intern Med. 2019;33(1):158–66. doi: 10.1111/jvim.15352 30499189 PMC6335532

[25] Curtis-Robles R, Zecca IB, Roman-Cruz V, Carbajal ES, Auckland LD, Flores I, et al. *Trypanosoma cruzi* (agent of Chagas disease) in sympatric human and dog populations in "Colonias" of the Lower Rio Grande Valley of Texas. Am J Trop Med Hyg. 2017;96(4):805–14. doi: 10.4269/ajtmh.16-0789 28167589 PMC5392625

[26] Nieto PD, Boughton R, Dorn PL, Steurer F, Raychaudhuri S, Esfandiari J, et al. Comparison of two immunochromatographic assays and the indirect immunofluorescence antibody test for diagnosis of *Trypanosoma cruzi* infection in dogs in south central Louisiana. Vet Parasitol. 2009;165(3–4):241–7. doi: 10.1016/j.vetpar.2009.07.010 19647943

[27] Meyers AC, Meinders M, Hamer SA. Widespread *Trypanosoma cruzi* infection in government working dogs along the Texas-Mexico border: Discordant serology, parasite genotyping and associated vectors. PLoS Negl Trop Dis. 2017;11(8):e0005819. doi: 10.1371/journal.pntd.0005819 28787451 PMC5560752

[28] Curtis-Robles R, Snowden KF, Dominguez B, Dinges L, Rodgers S, Mays G, et al. Epidemiology and molecular typing of *Trypanosoma cruzi* in naturally-infected hound dogs and associated triatomine vectors in Texas, USA. PLoS Negl Trop Dis. 2017;11(1):e0005298. doi: 10.1371/journal.pntd.0005298 28095511 PMC5287457

[29] Tenney TD, Curtis-Robles R, Snowden KF, Hamer SA. Shelter dogs as sentinels for *Trypanosoma cruzi* transmission across Texas. Emerg Infect Dis. 2014;20(8):1323–6. doi: 10.3201/eid2008.131843 25062281 PMC4111162

[30] Meyers AC, Hamer SA, Matthews D, Gordon SG, Saunders AB. Risk factors and select cardiac characteristics in dogs naturally infected with *Trypanosoma cruzi* presenting to a teaching hospital in Texas. J Vet Intern Med. 2019;33(4):1695–706. doi: 10.1111/jvim.15516 31119786 PMC6639495

[31] Meyers AC, Edwards EE, Sanders JP, Saunders AB, Hamer SA. Fatal Chagas myocarditis in government working dogs in the southern United States: Cross-reactivity and differential diagnoses in five cases across six months. Veterinary Parasitology: Regional Studies and Reports. 2021;24. doi: 10.1016/j.vprsr.2021.100545 34024363

[32] Vitt JP, Saunders AB, O’Brien MT, Mansell J, Ajithdoss DK, Hamer SA. Diagnostic features of acute Chagas myocarditis with sudden death in a family of boxer dogs. J Vet Intern Med. 2016;30(4):1210–5. doi: 10.1111/jvim.13967 27163180 PMC5084738

[33] Meyers AC, Purnell JC, Ellis MM, Auckland LD, Meinders M, Hamer SA. Nationwide exposure of U.S. working dogs to the Chagas disease parasite, *Trypanosoma cruzi*. Am J Trop Med Hyg. 2020;102(5):1078–85. doi: 10.4269/ajtmh.19-0582 32189615 PMC7204581

[34] Garcia MN, O’Day S, Fisher-Hoch S, Gorchakov R, Patino R, Feria Arroyo TP, et al. One health interactions of Chagas disease vectors, canid hosts, and human residents along the Texas-Mexico border. PLoS Negl Trop Dis. 2016;10(11):e0005074. doi: 10.1371/journal.pntd.0005074 27832063 PMC5104435

[35] Sarkar S, Strutz SE, Frank DM, Rivaldi CL, Sissel B, Sanchez-Cordero V. Chagas disease risk in Texas. PLoS Negl Trop Dis. 2010;4(10). doi: 10.1371/journal.pntd.0000836 20957148 PMC2950149

[36] Dumonteil E, Elmayan A, Majeau A, Tu W, Duhon B, Marx P, et al. Genetic diversity of *Trypanosoma cruzi* parasites infecting dogs in southern Louisiana sheds light on parasite transmission cycles and serological diagnostic performance. PLoS Negl Trop Dis. 2020;14(12):e0008932. doi: 10.1371/journal.pntd.0008932 33332357 PMC7775123

[37] Barbabosa-Pliego A, Diaz-Albiter HM, Ochoa-Garcia L, Aparicio-Burgos E, Lopez-Heydeck SM, Velasquez-Ordonez V, et al. *Trypanosoma cruzi* circulating in the southern region of the State of Mexico (Zumpahuacan) are pathogenic: a dog model. Am J Trop Med Hyg. 2009;81(3):390–5. 19706902 PMC2784919

[38] Piron M, Fisa R, Casamitjana N, Lopez-Chejade P, Puig L, Verges M, et al. Development of a real-time PCR assay for *Trypanosoma cruzi* detection in blood samples. Acta Trop. 2007;103(3):195–200. doi: 10.1016/j.actatropica.2007.05.019 17662227

[39] Duffy T, Cura CI, Ramirez JC, Abate T, Cayo NM, Parrado R, et al. Analytical performance of a multiplex Real-Time PCR assay using TaqMan probes for quantification of *Trypanosoma cruzi* satellite DNA in blood samples. PLoS Negl Trop Dis. 2013;7(1):e2000. doi: 10.1371/journal.pntd.0002000 23350002 PMC3547845

[40] Schijman AG, Bisio M, Orellana L, Sued M, Duffy T, Mejia Jaramillo AM, et al. International study to evaluate PCR methods for detection of *Trypanosoma cruzi* DNA in blood samples from Chagas disease patients. PLoS Negl Trop Dis. 2011;5(1):e931. doi: 10.1371/journal.pntd.0000931 21264349 PMC3019106

[41] Curtis-Robles R, Auckland LD, Hodo CL, Snowden KF, Nabity MB, Hamer SA. *Trypanosoma cruzi* discrete typing unit TcIV implicated in a case of acute disseminated canine Chagas disease. Vet Parasitol Reg Stud Reports. 2018;12:85–8. doi: 10.1016/j.vprsr.2018.02.006 31014814

[42] Curtis-Robles R, Hamer SA, Lane S, Levy MZ, Hamer GL. Bionomics and spatial distribution of triatomine vectors of *Trypanosoma cruzi* in Texas and other southern states, USA. Am J Trop Med Hyg. 2018;98(1):113–21. doi: 10.4269/ajtmh.17-0526 29141765 PMC5928729

[43] Gurtler RE, Cecere MC, Lauricella MA, Cardinal MV, Kitron U, Cohen JE. Domestic dogs and cats as sources of *Trypanosoma cruzi* infection in rural northwestern Argentina. Parasitology. 2007;134(Pt 1):69–82. doi: 10.1017/S0031182006001259 17032467 PMC2669415

[44] World Health O. Research priorities for Chagas disease, human African trypanosomiasis and leishmaniasis. World Health Organ Tech Rep Ser. 2012(975):v–xii, 1–100. 23484340

[45] Arce-Fonseca M, Carrillo-Sanchez SC, Molina-Barrios RM, Martinez-Cruz M, Cedillo-Cobian JR, Henao-Diaz YA, et al. Seropositivity for *Trypanosoma cruzi* in domestic dogs from Sonora, Mexico. Infect Dis Poverty. 2017;6(1):120. doi: 10.1186/s40249-017-0333-z 28870247 PMC5584529

[46] Machado EM, Fernandes AJ, Murta SM, Vitor RW, Camilo DJ Jr., Pinheiro SW, et al. A study of experimental reinfection by *Trypanosoma cruzi* in dogs. Am J Trop Med Hyg. 2001;65(6):958–65. doi: 10.4269/ajtmh.2001.65.958 11792006

[47] Tarleton RL, Reithinger R, Urbina JA, Kitron U, Gurtler RE. The challenges of Chagas Disease—grim outlook or glimmer of hope. PLoS Med. 2007;4(12):e332. doi: 10.1371/journal.pmed.0040332 18162039 PMC2222930

[48] Meyers AC, Ellis MM, Purnell JC, Auckland LD, Meinders M, Saunders AB, et al. Selected cardiac abnormalities in *Trypanosoma cruzi* serologically positive, discordant, and negative working dogs along the Texas-Mexico border. BMC Vet Res. 2020;16(1):101. doi: 10.1186/s12917-020-02322-6 32228593 PMC7106864

[49] Cecere MC, Vasquez-Prokopec GM, Gurtler RE, Kitron U. Reinfestation sources for Chagas disease vector, *Triatoma infestans*, Argentina. Emerg Infect Dis. 2006;12(7):1096–102. doi: 10.3201/eid1207.051445 16836826 PMC1853288

[50] Bello Corassa R, Aceijas C, Alves PAB, Garelick H. Evolution of Chagas’ disease in Brazil. Epidemiological perspective and challenges for the future: a critical review. Perspect Public Health. 2017;137(5):289–95. doi: 10.1177/1757913916671160 27758973

[51] Galaviz-Silva L, Mercado-Hernandez R, Zarate-Ramos JJ, Molina-Garza ZJ. Prevalence of *Trypanosoma cruzi* infection in dogs and small mammals in Nuevo Leon, Mexico. Rev Argent Microbiol. 2017;49(3):216–23. doi: 10.1016/j.ram.2016.11.006 28705488

[52] Gurtler RE, Lauricella M, Solarz ND, Bujas MA, Wisnivesky-Colli C. Dynamics of transmission of *Trypanosoma cruzi* in a rural area of Argentina. I-The dog reservoir: an epidemiological profile. Rev Inst Med Trop Sao Paulo. 1986;28(1):28–35. doi: 10.1590/s0036-46651986000100006 3094132

