# Supplementary material for: High incidence of Trypanosoma cruzi infections in dogs directly detected through longitudinal tracking at 10 multi-dog kennels, Texas, USA
Source: PLoS Negl Trop Dis. 2021 Nov 10;15(11):e0009935. doi: 10.1371/journal.pntd.0009935 (PMC8631682; doi:10.1371/journal.pntd.0009935)
Supplement: S1 Strobe Checklist — (DOCX) [file pntd.0009935.s001.docx]

STROBE Statement—Checklist of items that should be included in reports of ***cohort studies***

|  | Item No | Recommendation | Page No | Relevant text from manuscript |
| --- | --- | --- | --- | --- |
| **Title and abstract** | 1 | (*a*) Indicate the study’s design with a commonly used term in the title or the abstract | 1 | “Using a prospective cohort study, we monitored a cohort of 64 *T. cruzi*-infected and uninfected dogs from across 10 kennels in Texas, USA” |
|  |  | (*b*) Provide in the abstract an informative and balanced summary of what was done and what was found | 1 |  |
| Introduction | | | |  |
| Background/rationale | 2 | Explain the scientific background and rationale for the investigation being reported | 1-3 | “Direct measurements of incidence in natural animal populations are rare, as they require tracking of healthy, uninfected individuals over time to monitor for new infections.” “We used a longitudinal study design to directly measure incidence of *T. cruzi* infection in dogs of central/South Texas, an area with a high risk of Chagas disease based on suitable triatomine habitat and autochthonous human cases (36).” |
| Objectives | 3 | State specific objectives, including any prespecified hypotheses | 3 | “In this study, we tracked the serostatus and PCR-status of a matched cohort of *T. cruzi-*positive and *T. cruzi-*negative dogs at three time points over a 12-month period to monitor consistency or changes in infection status over time, allowing for the direct detection of an unprecedented incidence of *T. cruzi* infection in dogs in kennel environments.” |
| Methods | | | |  |
| Study design | 4 | Present key elements of study design early in the paper | 3 | “Using a prospective cohort study design, we enrolled a network of 10 multi-dog kennels throughout Central and South Texas with a prior history of *T. cruzi* infection in at least one of their dogs.” |
| Setting | 5 | Describe the setting, locations, and relevant dates, including periods of recruitment, exposure, follow-up, and data collection | 3 | ‘10 multi-dog kennels throughout Central and South Texas with a prior history of *T. cruzi* infection in at least one of their dogs. Dogs at these kennels are bred and trained primarily to aid hunting parties or compete in American Kennel Club dog events.” “During our initial sampling timepoint between May and July 2018” “The second sampling event occurred between December 2018 and March 2019, and the third sampling event occurred between May and September 2019.” “At each of the three timepoints, serum samples were tested for *T. cruzi* antibodies” |
| Participants | 6 | (*a*) Give the eligibility criteria, and the sources and methods of selection of participants. Describe methods of follow-up | 3 | “Enrollment criteria included dogs of any breed or sex at least one year of age, in residence at one of the selected kennels, and negative for other selected infectious diseases” “we performed serologic surveillance on a total of 134 dogs (between seven and 20 dogs at each of the 10 kennels) with a goal of identifying and enrolling approximately 2-4 *T. cruzi*-infected and 2-4 *T. cruzi*-uninfected dogs at each kennel for longitudinal tracking at approximate 6-month intervals” “All 10 kennels were visited at each timepoint to collect blood samples from enrolled dogs." |
|  |  | (*b*) For matched studies, give matching criteria and number of exposed and unexposed | 3 | “When possible, positive and negative dogs were frequency (group) matched on the basis of age, sex, and breed across all kennels” “From these dogs, a total of 64 dogs were enrolled in the study: 30 *T. cruzi-*positive and 34 *T. cruzi-*negative” |
| Variables | 7 | Clearly define all outcomes, exposures, predictors, potential confounders, and effect modifiers. Give diagnostic criteria, if applicable | 3-4 | “These two tests were run according to the manufacturers’ instructions, and a clear band developing after 15 minutes was considered positive. In the case that a very faint band developed, the test result was interpreted as negative (29). The IFA was performed by the Texas Veterinary Medical Diagnostic Laboratory (TVMDL) using 200 µL of serum to test for IgG antibodies against *T. cruzi*. Reactivity on at least two of the three serologic tests was required to enroll a dog as positive.” “An IDEXX 4Dx test (IDEXX Laboratories, Inc., Westbrook, ME) was run to exclude any dogs that may have had other parasitic infections” “a sample was considered positive if the CT value was under 36” |
| Data sources/ measurement | 8* | For each variable of interest, give sources of data and details of methods of assessment (measurement). Describe comparability of assessment methods if there is more than one group | 3-4 | “At each of the three timepoints,” “between May and July 2018” “between December 2018 and March 2019” “between May and September 2019” “serum samples were tested for *T. cruzi* antibodies using three serological tests: Chagas Stat-Pak (ChemBio, Medford, NY, USA), Trypanosoma Detect (InBios, International, Inc, Seattle, WA), and an indirect fluorescent antibody (IFA) test.” “We calculated the kappa index to determine the agreement among all three serological tests at each timepoint” “Samples were tested using qPCR for the presence of *T. cruzi* satellite DNA” |
| Bias | 9 | Describe any efforts to address potential sources of bias |  | “*T. cruzi-*positive and *T. cruzi-*negative dogs were frequency (group) matched on the basis of age, sex, and breed across all kennels.” |
| Study size | 10 | Explain how the study size was arrived at | 3 | “During the initial sampling time point between May and July 2018, we tested 134 dogs (between seven and 20 dogs at each of the 10 kennels) for evidence of *T. cruzi* and four other vector-borne infections. Based on the enrollment criteria, 64 dogs were identified as meeting the criteria and were enrolled in the study.” |
| Quantitative variables | 11 | Explain how quantitative variables were handled in the analyses. If applicable, describe which groupings were chosen and why | 4 | “Dogs were categorized as positive at a given timepoint if they met the criteria for serological positivity (positive on at least two of three independent serologic assays) and/or if they met the criteria for PCR positivity.” “various hound breeds were considered “Hound” for matching purposes” |
| Statistical methods | 12 | (*a*) Describe all statistical methods, including those used to control for confounding | 4 | “We calculated the kappa index to determine the agreement among all three serological tests at each timepoint (23, 29). To describe the risk of *T. cruzi* infection in the population of dogs that were initially enrolled as negative, we calculated the incidence rate as the number of new *T. cruzi* infections per 100 dogs per year.” “We used a Kaplan Meier survivor analysis to analyze the time a dog was enrolled as negative until conversion to positivity.” |
|  |  | (*b*) Describe any methods used to examine subgroups and interactions |  |  |
|  |  | (*c*) Explain how missing data were addressed |  |  |
|  |  | (*d*) If applicable, explain how loss to follow-up was addressed | 4 | “To accurately reflect the time negative dogs in the population were at risk, 0.5 years was subtracted from the population at risk (n=34) for each of the three negative dogs lost to follow up after the 6-month timepoint (n=3).” |
|  |  | (*e*) Describe any sensitivity analyses |  |  |
| Results | | |  |  |
| Participants | 13* | (a) Report numbers of individuals at each stage of study—eg numbers potentially eligible, examined for eligibility, confirmed eligible, included in the study, completing follow-up, and analysed | 3, 4-5 | “During the initial screening of 134 dogs “ “We enrolled 30 positive and 34 negative dogs across 10 kennels” “In total, five dogs were lost to follow up after the 6-month timepoint: two dogs enrolled as negative (including one that seroconverted to positive by 6-months) moved locations and were sold to new owners, and three dogs (two negative and one positive) had died.” |
|  |  | (b) Give reasons for non-participation at each stage | 5 | “During the initial screening…one dog was positive for *Dirofilaria immitis* and was excluded from the study” “two dogs enrolled as negative…moved locations and were sold to new owners, and three dogs…had died.” |
|  |  | (c) Consider use of a flow diagram |  |  |
| Descriptive data | 14* | (a) Give characteristics of study participants (eg demographic, clinical, social) and information on exposures and potential confounders | 3-4 | “30 positive and 34 negative dogs across 10 kennels in Central and South Texas” “Dogs enrolled in the study were between the ages of 13 months and 12.2 years (mean = 6.1 years; median = 6.1 years).” “average negative = 5.8 years; average positive = 6.6 years” “negative = 19 females and 15 males; positive = 14 males and 16 females” “3 positive and negative Belgian Malinois, 2 positive and negative German Shorthair Pointers, 3 positive and negative Hounds, 1 positive and 7 negative Labrador Retrievers, 2 positive and negative Brittany Spaniels, and 19 positive and 17 negative English Pointers” |
|  |  | (b) Indicate number of participants with missing data for each variable of interest |  |  |
|  |  | (c) Summarise follow-up time (eg, average and total amount) | 3 | One year follow-up time as follows: “…initial sampling time point between May and July 2018.” “The second sampling event occurred between December 2018 and March 2019, and the third sampling event occurred between May and September 2019. All 10 kennels were visited at each time point to collect blood samples from enrolled dogs.” |
| Outcome data | 15* | Report numbers of outcome events or summary measures over time | 5-6 | “Twenty-nine of the 30 dogs enrolled as positive maintained their positive status…across the study “ “Of the 34 dogs enrolled as negative, 24 (70.6%) remained negative at the 6-month and 12-month timepoints” “A minimum of eight out of the 34 (23.5%) dogs enrolled as negative converted to positive throughout the one-year study.” |

| Main results | 16 | (*a*) Give unadjusted estimates and, if applicable, confounder-adjusted estimates and their precision (eg, 95% confidence interval). Make clear which confounders were adjusted for and why they were included | 5-6 | “The Kappa statistics comparing serological tests at each sampling timepoint” “After adjusting the time that the total population was at risk to account for attrition of three negative dogs after the 6-months timepoint” “A Kaplan-Meier survival curve was generated to show the proportion of dogs enrolled as negative that converted to positive over the 12-month study” “The average 95% confidence interval” |
| --- | --- | --- | --- | --- |
|  |  | (*b*) Report category boundaries when continuous variables were categorized |  |  |
|  |  | (*c*) If relevant, consider translating estimates of relative risk into absolute risk for a meaningful time period |  |  |
| Other analyses | 17 | Report other analyses done—eg analyses of subgroups and interactions, and sensitivity analyses |  |  |
| Discussion | | | |  |
| Key results | 18 | Summarise key results with reference to study objectives | 6 | “We characterized the *T. cruzi* serologic and PCR-status of 64 dogs at 3 timepoints over the course of one year and demonstrated a high incidence of *T. cruzi* infection in a cohort of dogs housed in multi-dog kennels across Texas. In our study cohort of 34 initially negative dogs, we recorded 8-10 new infections (23.5-29.4%) resulting in an incidence rate of 24.6-30.7 new infections per 100 years.” |
| Limitations | 19 | Discuss limitations of the study, taking into account sources of potential bias or imprecision. Discuss both direction and magnitude of any potential bias | 7-8 | “There is no gold standard for diagnosing *T. cruzi* infections in dogs… and our study highlights the need…for improved diagnostic tools” “One limitation to this finding is the variation in incident cases across the 10 studied kennels.” |
| Interpretation | 20 | Give a cautious overall interpretation of results considering objectives, limitations, multiplicity of analyses, results from similar studies, and other relevant evidence | 6-8 | In reference to other cited studies, “we were able to track the same dogs over a period of one year to detect seroconversion and PCR conversion, and thus report a direct measure of incidence in a closed population of dogs |
| Generalisability | 21 | Discuss the generalisability (external validity) of the study results | 8 | “Multi-dog kennels should be areas of targeted intervention in the US, as the incidence of dog infection is high.” |
| Other information | | | |  |
| Funding | 22 | Give the source of funding and the role of the funders for the present study and, if applicable, for the original study on which the present article is based | 8 | “The American Kennel Club Canine Health Foundation Grant No 02448 provided funding.” |

*Give information separately for exposed and unexposed groups.

**Note:** An Explanation and Elaboration article discusses each checklist item and gives methodological background and published examples of transparent reporting. The STROBE checklist is best used in conjunction with this article (freely available on the Web sites of PLoS Medicine at http://www.plosmedicine.org/, Annals of Internal Medicine at http://www.annals.org/, and Epidemiology at http://www.epidem.com/). Information on the STROBE Initiative is available at http://www.strobe-statement.org.
